# The role of soluble toll-like receptor-2 and 4 in children with pneumonia: a combined analysis of saliva and serum samples

**DOI:** 10.3389/fimmu.2026.1657027

**Published:** 2026-02-13

**Authors:** Ozlem Necipoglu, Osman Oguz Demir, Kubra Aykac, Sevilay Karahan, Ali Bulent Cengiz, Malik Aydin, Yasemin Ozsurekci

**Affiliations:** 1 Department of Pediatrics, Hacettepe University Faculty of Medicine, Ankara, Türkiye; 2 The Hospital for Sick Children Research Institute, Cell Biology Research Program, Toronto, ON, Canada; 3 Department of Pediatric Infectious Diseases, Hacettepe University Faculty of Medicine, Ankara, Türkiye; 4 Department of Biostatistics, Hacettepe University Faculty of Medicine, Ankara, Türkiye; 5 Laboratory of Translational Medicine and Pediatric Infectious Diseases, Center for Biomedical Education and Research (ZBAF), Department of Human Medicine, Faculty of Health, Witten/Herdecke University, Witten, Germany; 6 Virology and Microbiology, Center for Biomedical Education and Research (ZBAF), Faculty of Health, Department of Human Medicine, Witten/Herdecke University, Witten, Germany; 7 Chair of Pediatrics, Vestische Kinder- und Jugendklinik Datteln, Witten/Herdecke University, Datteln, Germany

**Keywords:** cytokine, disease severity, lower respiratory tract infection, pediatrics, SARS-CoV-2

## Abstract

**Background:**

Since rapid and accurate diagnosis of pneumonia and determination of its severity remain challenging, particularly in childhood, we aimed to evaluate the role of toll-like receptors (TLR) in pneumonia, which has been limited in animal and adult studies.

**Methods:**

In this study, TLR-2, TLR-4, interleukin (IL)-1β, IL-4, IL-6, IL-10, interferon (IFN)-α, and tumor necrosis factor (TNF)-α levels were measured in serum samples of *n* = 67 pediatric patients with *community acquired pneumonia (CAP)* (43 inpatients and 24 outpatients) and 22 healthy pediatric controls. Saliva samples of *n* = 30 pediatric patients with CAP (19 inpatients and 11 outpatients) and 19 healthy pediatric controls were also investigated to analyze the expression of these cytokines and TLRs.

**Results:**

In saliva samples taken at the time of diagnosis, threshold levels of TLR-2 were 1.16 2^-ΔΔ^*^Ct^* and of TLR-4 1.28 2^-ΔΔ^*^Ct^* differentiating patients with and without pneumonia. The sensitivity of salivary TLR-2 and TLR-4 tests were found to be 0.80 and 0.86, respectively. In serum samples, soluble TLR-2 and TLR-4, IL-1β, IL-4, IL-6, Il-10, IFN-α, and TNF-α levels were significantly decreased in patients with coronavirus disease-19 (COVID-19) as compared with other viruses (*p* < 0.05 in each). Only one mortal case was present in the inpatient group during the study period.

**Conclusion:**

This study demonstrates that salivary TNF-α, soluble TLR-2 and TLR-4 may serve as promising non-invasive biomarkers for diagnosing and stratifying pediatric pneumonia.

## 1 Introduction

Pneumonia is an important cause of death in children worldwide. According to the data of the World Health Organization (WHO) in 2017, 808.694 child deaths caused by pneumonia, which constituted 15% of all deaths under the age of five (1). Few factors playing a role in the inflammatory response in the pathogenesis of pneumonia provided some clues about the morbidity of the disease and the intensity of inflammation (2, 3).

Although data on community acquired pneumonia (CAP) in children is increasing, several information in both diagnosis and treatment in childhood are still missing (4). Identifying the causative agent or trigger of pneumonia in children could be more challenging than in adults due to difficulties in obtaining representative lower respiratory specimens, blood volumes, and limitations in applying invasive diagnostics (5). An accurate risk assessment is thus required to minimize unnecessary diagnostic testing, treatments, and hospitalizations while focusing on critical treatments for those at highest risk for serious outcomes. However, few objective prognostic tools are currently available for children with CAP. Without such tools, clinical management for children with CAP may have potential to be inefficient (6). Studies on several biomarkers including cytokines that may be associated with inflammatory processes in children receiving treatment for pneumonia have been already performed (7, 8). Classical biomarkers, such as serum procalcitonin (PCT) and C-reactive protein (CRP) have been used frequently in the diagnosis of severe pneumonia (8–11). In addition, many markers e.g., white blood cell count (WBC), neutrophil and lymphocyte counts, PCT, and CRP have been also investigated to understand causative reasons of pneumonia caused by bacteria and viruses, however there is still no valid diagnostic biomarker for an efficient management of pneumonia for physicians (12–14).

Although it has been determined that toll-like receptors (TLRs) have been studied in several animal experiments and human studies, especially in children are limited (15, 16). As a result of binding of ligands to cells, such as macrophages, dendritic cells, and mast cells, TLR-2, which is found as transmembrane protein in these cells, undergoes dimerization with its co-domains, known as TLR-1 and TLR-6, causing activation of some signaling pathways (17). In particular, LPS components of the cell walls of gram-negative bacteria may trigger the intracellular inflammation network through TLR-4 and initiate the proinflammatory process (17–19). The interaction of the SARS-CoV-2 envelope (E) protein with TLR-2 is among the events that initiate a pro-inflammatory process, in which inflammatory cytokines, including tumor necrosis factor (TNF)-α and interferon (IFN)-γ are released (20). Specific peptides have also been identified as TLR-2 agonists within the spike protein. While these proteins typically induce the signaling through MyD88 pathways, they also cause TLR-2 to be taken into the cell and endosomes, subsequently inducing the type I/III IFN response (21, 22).

Soluble forms of TLRs (sTLRs) are released into biological fluids through proteolytic shedding of membrane-bound receptors. These soluble forms can bind pathogen-associated molecular patterns (PAMPs) and prevent them from activating cell-surface TLRs, thereby functioning as decoy receptors that modulate inflammatory responses (23). TLR-2 has been found in serum, saliva, and other mucosal secretions, where it appears to reduce both bacterial and viral-induced cellular activation without impairing pathogen clearance (24–26). Studies have shown that sTLR-2 levels are associated with disease severity in various infectious diseases and can help distinguish infectious from non-infectious inflammation (24, 25, 27). This may be particularly relevant in pediatric respiratory infections, where children often show age-specific disease patterns. For example, while most children with COVID-19 experience mild disease, the interaction between SARS-CoV-2 proteins and TLR-2 (20, 21) raises the question of whether sTLR-2 levels might influence clinical severity in those who do develop severe manifestations.

In the context of pediatric CAP, this balance is particularly relevant. Effective pathogen clearance requires adequate immune activation, yet excessive inflammation may lead to significant lung injury. Both sTLR-2 and sTLR-4 may therefore function not only as immunomodulatory factors but also as potential biomarkers of disease severity. By measuring these soluble receptors in both saliva and serum, we can gain complementary insights into the local airway response as well as systemic immune activation. Therefore, to address the clinical and scientific relevance, we aimed to study the role of soluble TLR and cytokine responses in terms of disease severity of pediatric patients with CAP evaluating soluble TLR-2 and TLR-4 in both saliva and serum from children with pneumonia.

## 2 Materials and methods

### 2.1 Study design and participants

This study is a single-center and prospective case-control study, which was performed between July 2020 and July 2021. Pediatric patients aged 1 month to 18 years who were diagnosed with CAP due to their clinical and radiological features and who applied to tertiary care university hospital for children were recruited, and newborns were not included in the study. All consecutive patients meeting inclusion criteria during the study period were approached for enrollment. Community-acquired pneumonia was determined as written in the guidelines, including ‘2014 Revised WHO Classification and Treatment of Childhood Pneumonia at Health Facilities’ (28), ‘British Thoracic Society guidelines for the management of community acquired pneumonia in children: update 2011’ (29), and ‘Clinical Practice Guidelines by the Pediatric Infectious Diseases Society and the Infectious Diseases Society of America’ (30). Respiratory virus and bacteria multiplex PCR panel tests, blood culture, and pleural fluid cultures (if available) were used for the detection of the causative pathogen. Patients with cystic fibrosis, primary ciliary dyskinesia, immunodeficiency, systemic rheumatologic disease, leukemia, patients receiving chemotherapy, and patients using immunomodulatory agents were excluded from the study.

Patients with PCR-confirmed SARS-CoV-2 pneumonia were included in the study regardless of the presence of underlying chronic conditions. However, children with non-pulmonary systemic involvement of COVID-19 were not included in the study. Furthermore, patients with COVID-19-associated multisystem inflammatory syndrome in children (MIS-C) were not included due to the accompanying inflammatory processes that have not yet been elucidated. In the patient group, 67 serum samples (*n* = 43 inpatients and *n* = 24 outpatients) and 30 saliva samples (*n* = 19 inpatients and *n* = 11 outpatients) were included (**Supplementary Figure 1**). The saliva samples of the cases with pneumonia caused by SARS-CoV-2 could not be recovered due to the strict infection control measures at the university hospital during the previous SARS-CoV-2/COVID-19 pandemic. All samples taken from the patient group were obtained at the time of diagnosis. The age and gender matched healthy controls did not have active infection or inflammation, and did not use any medication. The control group consisted of 22 healthy children, of whom 21 provided serum samples and 19 provided saliva samples. An informed consent was obtained from parents and/or legal guardians of all participants in the patient and control groups, as well as from the participants aged 16 and over.

### 2.2 Serum analysis

The analyses of interleukin (IL)-1β, IL-4, IL-6, IL-10, IFN-α, TNF-α cytokines, soluble TLR-2, and TLR-4 in serum samples were performed using Bioassay Technology Laboratory (BT-Lab) sandwich Enzyme Linked Immunosorbent Assay (ELISA) kits with the respective catalog number was E0143Hu, E0092Hu, E0090Hu, E0076Hu, E0102Hu, E0082Hu, E0346Hu and E0358Hu according to manufacturer’s instructions. This process was done separately for each 8 markers in serum.

### 2.3 Saliva analysis

The protocol for RNA to cDNA in saliva is presented in **Supplementary Table 1A**. Gene-specific primers for β-Actin (ACTB), TLR-2, TLR-4, IL-1B, IL-4, IL-6, IL-10, TNF-α, and IFN-α were designed with Primer-BLAST (NCBI) and synthesized by Oligomer (Ankara, Turkey). Real-time RT-PCR was performed with the SensiFAST™ SYBR No-ROX Kit (Bioline, Cat. No. BIO-98050, UK), following the manufacturer’s protocol. ACTB was used as a reference gene. Relative mRNA abundance was calculated by the 2^-ΔΔ^*^Ct^* method. Primer sequences are listed in **Supplementary Table 1B**.

### 2.4 Statistical analyses

Statistical analyses were performed and graphical images were created using IBM^®^ SPSS^®^ for Windows Version 23.0 and RStudio version 2024.04.1 + 748. Numerical variables were shown as mean ± standard deviation or median [25-75th percentile]. Categorical variables were stated as frequencies and percentages. Categorical variables were compared with either Chi-square or the Fisher exact tests. The normality of continuous variables was evaluated with the Kolmogorov Smirnov test, and the homogeneity of variances was tested using Levene’s test. Differences between groups including continuous variables were determined by Mann Whitney U- or Kruskal-Wallis tests, and for multiple comparisons, Bonferroni correction was applied. For each serum cytokine, the effects of age groups (0–1 yr, 1–5 yr, and 5–18 yr) and cohorts (Control/Inpatient/Outpatient) were tested with a two-way analysis of variance. Model assumptions, including normality and homoscedasticity of residuals, were assessed graphically. If diagnostic plots indicated deviations from model assumptions, results were confirmed using an aligned-rank transform ANOVA implemented through the *ARTool* package in R. Pair-wise contrasts were performed with Tukey’s honestly significant difference (HSD) test, and all *p*-values reported are Tukey-adjusted. Pairwise Pearson (continuous-continuous) or point-biserial (continuous-binary) correlations were calculated between quantitative laboratory indices, the serum and salivary markers concentrations. Receiver-operating characteristic (ROC) curve analysis was used to determine the diagnostic accuracy of the biomarkers. Sensitivity, specificity, negative, and positive predictive, as well as accuracy values were stated for these optimal cut-offs which was determined by using Index of Union and Youden’s Index. A *p-*value less than 0.05 was considered significant.

## 3 Results

### 3.1 Clinical characteristics of the patients with pneumonia

The median age of the hospitalized patients was 28 months (IQR, 12-89 months) and the outpatients 41.5 months (IQR, 18.25- 87). In the control group, the median age was 68 months (IQR, 52.75- 127.5) and there was no marked difference between these three groups (*p* = 0.069). Among the patients with pneumonia, 28 (65.1%) of the hospitalized patients were male and 15 (34.9%) female, while among the non-hospitalized patients, 19 (79.2%) were male and 5 (20.8%) female (*p* = 0.116) (**Table 1**).

**Table 1:** Clinical features of patients with pneumonia and healthy children.

Features	Inpatient (*n* = 43)	Outpatient (*n* = 24)	Control (*n* = 22)	*P*-value
Age, month (IQR)	28 (12-89)	41.5 (18.25-87)	68 (52.75-127.5)	0.069
Gender				0.116
Male (n, %)	28 (65.1)	19 (79.2)	11 (50.0)	
Female (n, %)	15 (34.9)	5 (20.8)	11 (50.0)	
Symptoms				
Fever (n, %)	24 (55.8)	8 (33.3)	NA	0.131
Sore throat (n, %)	4 (9.3)	4 (16.7)	NA	0.443
Cough (n, %)	27 (62.8)	20 (83.3)	NA	0.138
Increased mucus production (n, %)	21 (48.8)	10 (41.7)	NA	0.757
Runny nose (n, %)	8 (18.6)	12 (50.0)	NA	0.016*
Wheezing (n, %)	18 (41.9)	9 (37.5)	NA	0.929
Respiratory distress (n, %)	28 (65.1)	7 (29.2)	NA	0.010*
Tachypnea (n, %)	23 (53.5)	6 (25)	NA	0.046*
Fatigue (n, %)	10 (23.3)	2 (8.3)	NA	0.188
Anorexia (n, %)	10 (23.3)	3 (12.5)	NA	0.350
Feeding intolerance (n, %)	11 (25.6)	3 (12.5)	NA	0.342
Vomiting (n, %)	9 (20.9)	4 (16.7)	NA	0.757
Antibiotic use before admission (n, %)	11 (25.6)	1 (4.2)	NA	0.044*
Days from symptom onset to admission (Min-max)	2 (0-15)	2 (0-15)	NA	0.808
Chronic disease				<0.001***
None (n, %)	18 (41.9)	8 (33.3)	22 (100)	NA
Cardiac diseases (n, %)	2 (4.7)	2 (8.3)	0	NA
Neurologic diseases (n, %)	15 (34.9)	5 (20.8)	0	NA
Pulmonary diseases (n, %)	4 (9.3)	1 (4.2)	0	NA
Metabolic diseases (n, %)	2 (4.7)	0 (0.0)	0	NA
Skeletal disorders (n, %)	1 (2.3)	1 (4.2)	0	NA
Allergic diseases (n, %)	1 (2.3)	6 (25)	0	NA
Gastrointestinal diseases (n, %)	0 (0.0)	1 (4.2)	0	NA
History of recurrent respiratory infections (n, %)	18 (41.9)	12 (50)	NA	0.699
Tracheostomy (n, %)	5 (11.6)	1 (4.2)	NA	0.408
Gastrostomy (n, %)	3 (7.0)	1 (4.2)	NA	1.000
PICU admission (n, %)	11 (25.6)	0 (0.0)	NA	NA
Viral pneumonia (n, %)	30 (69.8)	15 (62.5)	NA	NA
Bacterial pneumonia (n, %)	3 (7.0)	0 (0.0)	NA	NA
Pneumonia with unknown pathogen (n, %)	10 (23.2)	9 (37.5)	NA	NA
Mortality (n, %)	1 (2.3)	0 (0.0)	NA	NA

Statistical significance is indicated as follows: (***) for p < 0.001 and (*) for p < 0.05. NA, Not Applicated; PICU, pediatric intensive care unit.

Comparing inpatient and outpatient groups in terms of symptoms present at admission to the hospital, fever was more common in the inpatient group, but no statistically significant difference was found (55.8% and 33.3%, respectively, *p* = 0.131). Additional symptoms are summarized in **Table 1**. Running nose was more common in the outpatient group compared to the inpatient group (50.0% and 18.6%; *p* = 0.016). Patients with respiratory effort and distress at the time of admission were encountered more frequently in the inpatient group (65.1% and 29.2%; *p* = 0.010). Tachypnea was more present in *n* = 23 (53.5%) of the hospitalized patients and *n* = 6 (25%) of the outpatients (*p* = 0.046) (**Table 1****).**

The time required to evaluate antibiotic use prior to admission was accepted as one week before admission. When patient groups were compared, the use of antibiotics was more frequent in the inpatient group, and 11 patients (25.6%) from the inpatient and only one patient (4.2%) from the outpatient group received antibiotics (*p* = 0.044). The median time from symptom onset to admission was two days in both patient groups, with the longest as 15 days (*p* = 0.808). Underlying comorbidities (predominantly neurological (34.9% inpatients, 20.8% outpatients) and pulmonary diseases) as well as the presence of tracheostomy and gastrostomy, varied between patient groups, with detailed distributions presented in **Table 1**.

Respiratory tract viral and bacterial panel, blood, and pleural fluid culture results were useful in determining the causative pathogen. Thirty (69.8%) inpatients and 15 (62.5%) outpatients were diagnosed with viral pneumonia. Eleven patients (25.6%) from the inpatient group were admitted to the pediatric intensive care unit following admission. One of 43 hospitalized patients died, and the remaining 42 patients (97.7%) were discharged in good health (**Table 1****).**

### 3.2 Laboratory findings

The median CRP of the inpatient group was 2.65 mg/dL (IQR, 0.7-8.3 mg/dL), and of the outpatient group 0.71 mg/dL (IQR, 0.51-1.33 mg/dL) (*p* = 0.003). When levels of PCT at presentation were evaluated, the median value of the inpatient group was 0.15 ng/mL (IQR, 0.06-0.95 ng/mL) and of the outpatient group 0.07 ng/mL (IQR, 0.04 -0.10 ng/mL) (*p* = 0.007). There was no significant difference in terms of hemoglobin, leukocyte, lymphocyte, thrombocyte, NLR, and ESR values of the patients (**Supplementary Table 2****).** Among in-patients, 18 of 43 children with a history of recurrent LRTI were ≤ 5 years old (83.3%), and there were no routine laboratory indices or lymphocyte subsets differed between children with and without recurrence (**Supplementary Table 3****).**

### 3.3 Serum analyses

**Figure 1:**
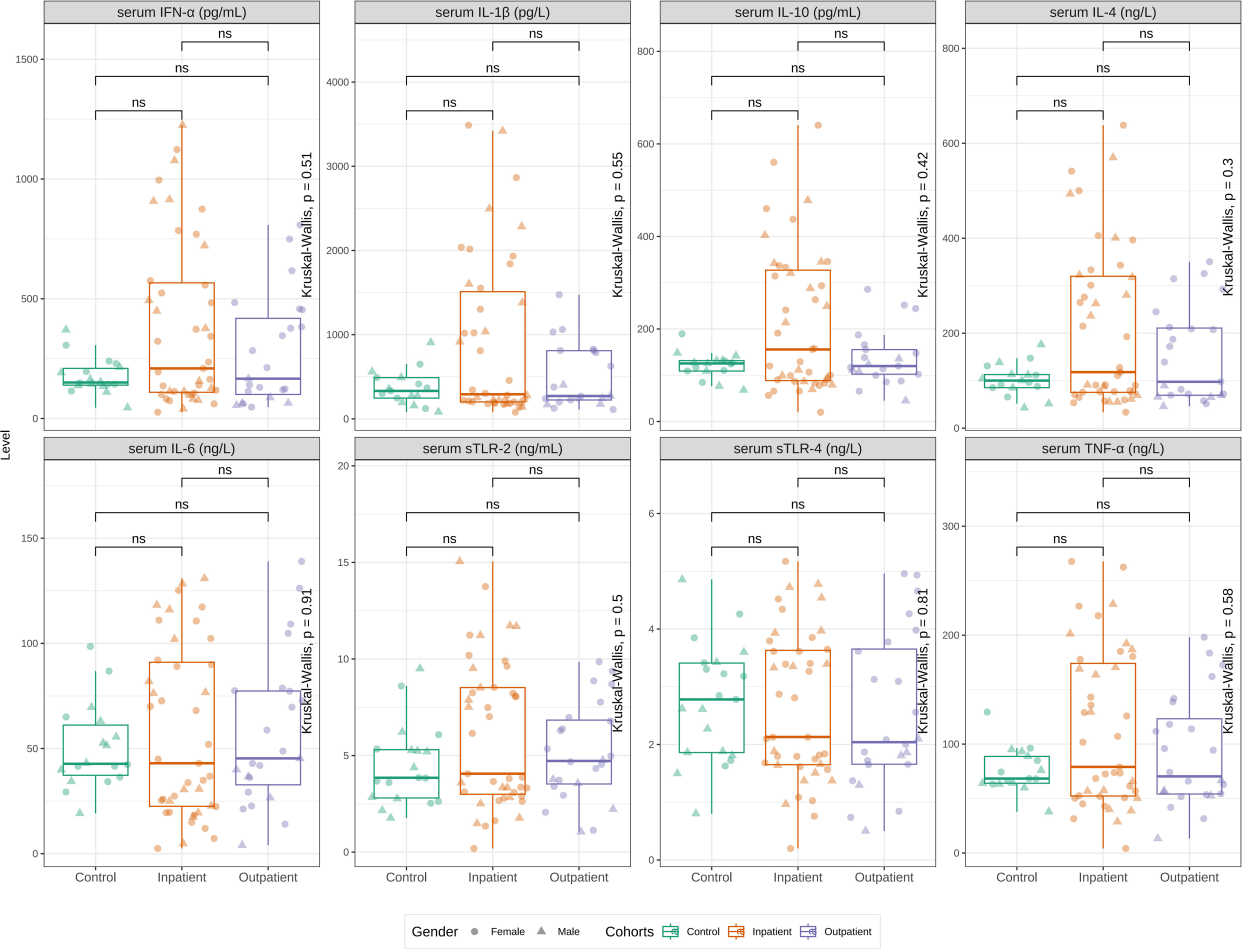
The comparison of TNF-α, IL-1β, IL-4, IL-6, IL-10, IFN-α, soluble TLR-2 and TLR-4 levels in serum samples of patients with pneumonia who were hospitalized (inpatients) and not hospitalized (outpatients) and healthy children (controls). There were no significant difference between groups.

Cohort-level comparisons of serum cytokines without age stratification demonstrated no significant differences among in- and outpatients, as well as controls (**Figure 1**) (all *p* > 0.05). As shown in **Supplementary Figure 1**, after stratifying by age (0–1, 1–5, and 6–18 years), a two-way ANOVA (age group × cohort) revealed significant age effects in seven cytokines, including IL-1β, IL-4, IL-6, IL-10, TNF-α, IFN-α, and soluble TLR-4 (all Tukey-adjusted *p* < 0.05). *Post hoc* tests indicated particularly strong age gradients within the inpatient cohort: infants (0 to 1 year) had higher levels than 6 to 17-year inpatients for IL-6 (*p* < 0.01), TNF-α (*p* < 0.001), IFN-α (*p* < 0.01), IL-1β (*p* < 0.01), IL-4 (*p* < 0.01), IL-10 (p < 0.001), and TLR-4 (*p* < 0.01). For IL-6, both 1–5 and 6–18 years were lower than infants (both *p* < 0.05). In controls, TLR-2 levels were lower in older children (1 to 5 and 6 to 18 years) than in infants (both *p* < 0.05). Notably, when comparing same-age groups across cohorts (e.g., 6–18-year inpatients vs 6 to 18-year controls), no cytokine differed significantly (all Tukey-adjusted *p* > 0.05), consistent with the pooled overview in **Figure 1**.

**Figure 2:**
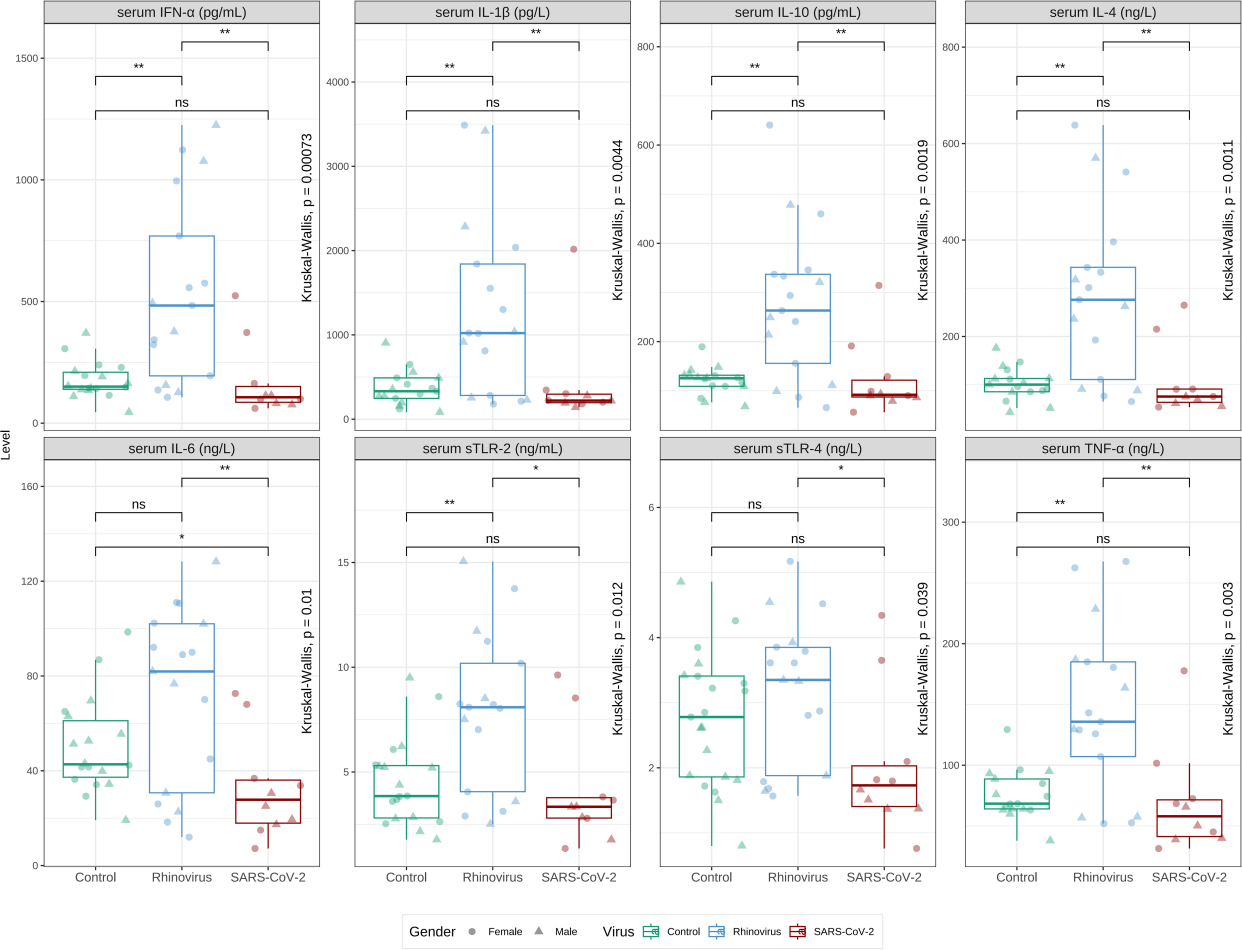
The comparison of serum markers among healthy children and hospitalized children with viral pneumonia caused by SARS-CoV-2 and human rhinovirus. All markers were significantly different between the groups (Kruskal-Wallis test) and between inpatients with SARS-CoV-2 and those with human rhinovirus. The reduction of markers in inpatients with SARS-CoV-2 was not significant except for serum IL-6, while the elevation of markers in inpatients with human rhinovirus was significant except for serum IL-6 and soluble TLR-4 when compared to healthy controls. Statistical significance is indicated as follows: (**) for *p* < 0.01, (*) for *p* < 0.05, and (ns) for *p* ≥ 0.05.

Among the 67 patients with pneumonia, viral pathogens were identified in 45 cases (67%). Human rhinovirus (*n* = 20; 13 hospitalized) and SARS-CoV-2 (*n* = 17; 9 hospitalized) were the predominant viruses detected during the study period, comprising 82% of viral cases. When cytokines and soluble TLRs in serum samples were evaluated, there was no significant difference between patients with pneumonia in which viruses were detected as the causative pathogen, and patients with pneumonia in whom the causative agent cannot be detected. However, virus-specific subgroup analyses revealed distinct immunological patterns between different viral etiologies. In particular, human rhinovirus and SARS-CoV-2 infections demonstrated relevant different immune profiles (**Figure 2**). Human rhinovirus pneumonia was characterized by broad immune activation, with significant elevation of IFN-α, IL-1β, IL-4, IL-10, TNF-α, TLR-2, and TLR-4 compared to controls (all p<0.05). In contrast, SARS-CoV-2 pneumonia showed a selective response pattern: while IL-1β, IL-4, IL-10, and TNF-α were elevated, IFN-α, IL-6, and TLR-4 levels were significantly lower than controls, distinguishing SARS-CoV-2 from typical viral respiratory infection patterns. The measured biomarkers differed significantly between human rhinovirus and SARS-CoV-2 groups, suggesting virus-specific immune pathways in pediatric pneumonia (**Figure 2****).**

### 3.4 Saliva analyses

IL-1β, IL-6, IL-10, IFN-α, TNF-α, TLR-2, and TLR-4 were analyzed in saliva samples using qPCR. In addition, IL-4 expression in saliva was below the limit of reliable detection in the majority of samples; therefore it was not included to the analyses.

Salivary TLR-2 expression (2^-ΔΔCt^) was significantly elevated in inpatients (median 3.81, IQR 2.09-5.45) compared to controls (median 0.98, IQR 0.54-1.86; *p* = 0.002), with outpatients showing intermediate levels (median 2.16, IQR 0.78-7.87). A similar pattern was observed for TLR-4, with inpatients demonstrating higher expression (median 4.26, IQR 2.52-6.51) than controls (median 0.97, IQR 0.57-1.81; p=0.001). These findings represent approximately 4-fold elevation in both TLR-2 and TLR-4 expression in severe pneumonia compared to healthy children.

Salivary cytokines also showed differential expression patterns. Pro-inflammatory cytokines IL-6, IL-10, IFN-α, and TNF-α were all significantly elevated in inpatients compared to controls (all *p* < 0.02), with TNF-α showing the most pronounced difference (>10-fold increase; median 10.34 vs 0.96; *p* < 0.001). Outpatients generally demonstrated intermediate expression levels between inpatients and controls, though this gradient was less consistent than observed for TLRs. However, the level of elevation for TNF-α was higher than for other measured cytokines.

**Figure 3:**
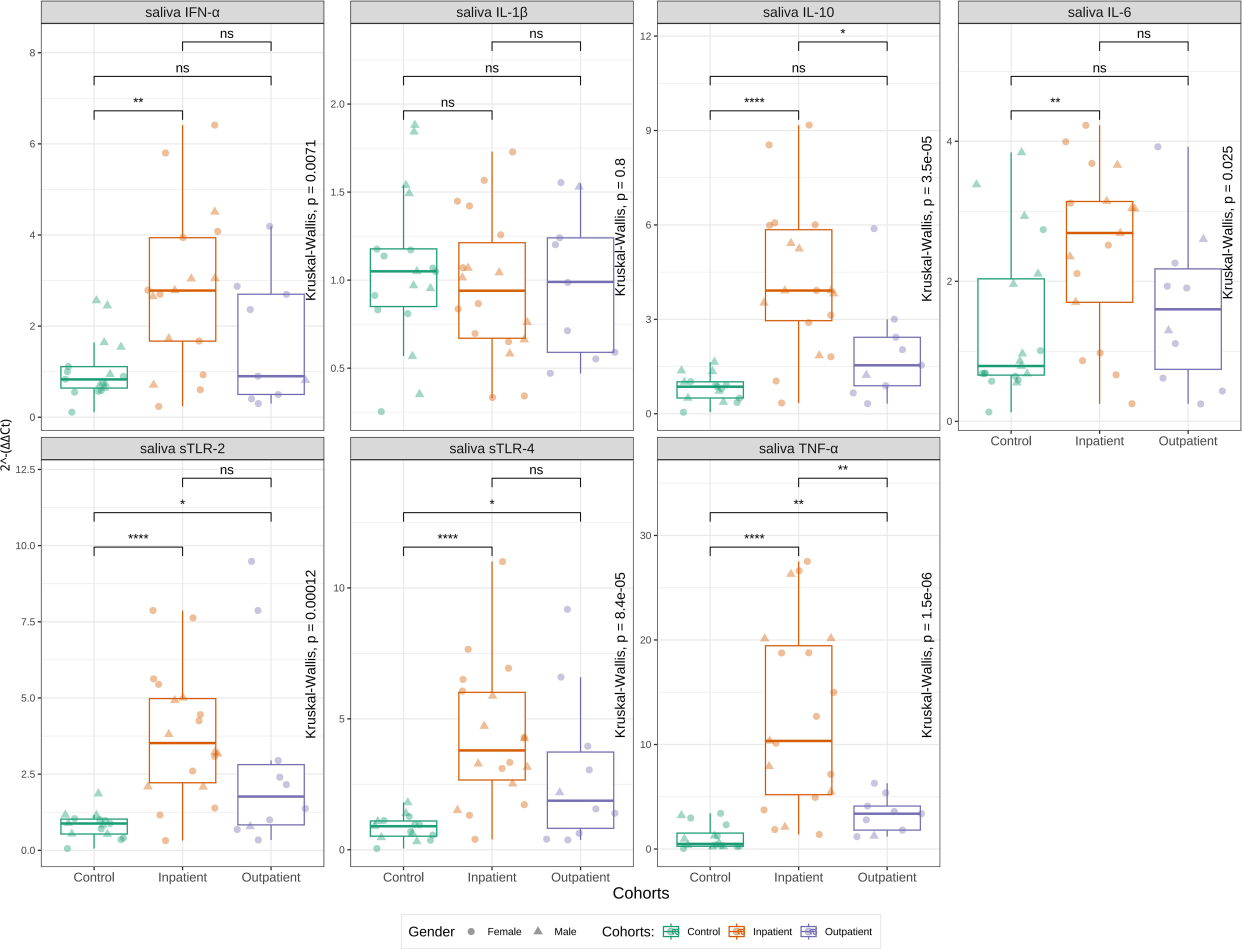
The comparison of TNF-α, IL-1β, IL-4, IL-6, IL-10, IFN-α, soluble TLR-2, and TLR-4 levels in saliva samples of patients with pneumonia (excluding COVID-19) who were inpatients and outpatients, and controls. In distinguishing these groups, all markers except for saliva IL-1β were significantly different (Kruskal-Wallis test). The high 2^-ΔΔ^*^Ct^* at all markers apart from IL-6 of inpatients had significant difference when compared control group. Statistical significance is indicated as follows: (****) for *p* < 0.0001, (**) for *p* < 0.01, (*) for *p* < 0.05, and (ns) for *p* ≥ 0.05.

The consistent pattern of elevated expression in inpatients compared to controls across all biomarkers suggests coordinated activation of both innate immune recognition (TLRs) and inflammatory response (cytokines) in severe pediatric pneumonia. Complete age-stratified data are presented in **Figure 3** and **Supplementary Figures 2**, **3**.

### 3.5 ROC analysis

**Figure 4:**
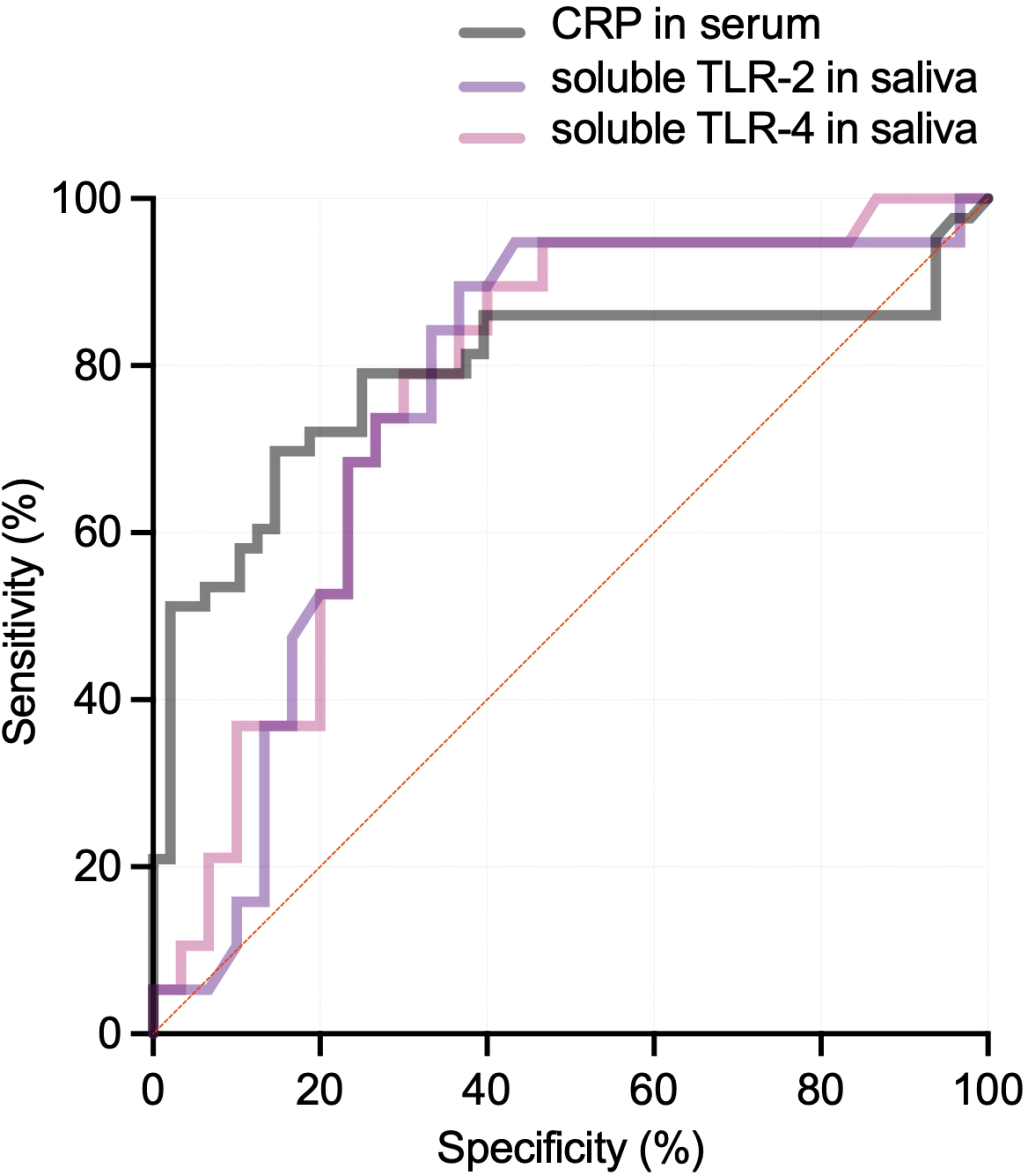
ROC curves for predicting inpatients with soluble TLR-2, soluble TLR-4 in saliva, and CRP levels in serum. The ROC curves indicate the predictive power of soluble TLR-2 with the AUC of 0.757 (95% CI, 0.615-0.899; *p* = 0.003), soluble TLR-4 with the AUC of (0.768 95% CI, 0.632-0.903; *p* = 0.002), and CRP with the AUC of (0.790 95% CI, 0.687-0.893; *p* = 0.001) for identifying hospitalized children with pneumonia.

Differentiating CAP that necessitating hospitalization across all cohorts, the areas under the ROC curves for saliva samples were as follows: soluble TLR-2 had an AUC of 0.757 (95% CI, 0.615-0.899; *p* = 0.003) with the optimal threshold at 1.970 2^-ΔΔ^*^Ct^*, soluble TLR-4 had an AUC of 0.768 (95% CI, 0.632-0.903; *p* = 0.002) with the optimal threshold at 2.350 2^-ΔΔ^*^Ct^*, and TNF-α had an AUC of 0.833 (95% CI, 0.721-0.946; *p* < 0.0001) with optimal thresholds at 3.665 2^-ΔΔ^*^Ct^*. An AUC of 0.833 for TNF-α indicates excellent predictive accuracy, meaning that there is an 83.3% chance that a randomly selected patient, who required hospitalization for CAP, will have a higher TNF-α level in saliva than a patient, who did not require hospitalization. This suggests that salivary TNF-α has strong predictive power for identifying more severe cases of CAP. In addition, among salivary markers, IL-6 had the highest specificity (76.7%, 95% CI 59.1–88.2; AUC 0.742, 95% CI 0.597–0.888; *p* = 0.005). Conversely, IL-1β showed the lowest sensitivity (47.4%, 95% CI 27.3–68.3) with a non-significant AUC of 0.565 (95% CI 0.400–0.730; *p* = 0.45). IFN-α yielded the lowest PPV and NPV (AUC 0.719, 95% CI 0.567–0.871; *p* = 0.01). At the optimum cut-off values determined by index of union and Youden’s index, the performance metrics for these biomarkers were as follows: for soluble TLR-2, the sensitivity was 84.2%, specificity was 66.7%, positive predictive value (PPV) was 61.5%, and negative predictive value (NPV) was 76.0%. For soluble TLR-4, the sensitivity was 79.0%, specificity was 70.0%, PPV was 62.5%, and NPV was 78.7%; and for TNF-α, the sensitivity was 84.2%, specificity was 73.3%, PPV was 66.7%, and NPV was 81.3% (**Figure 4**). When the same prediction with ROC analysis for hospitalized patients was performed across all cohorts, there were no significantly differentiating markers for serum biomarkers (**Table 2****).**

**Table 2:** ROC analysis and performance metrics for serum and saliva cytokines and soluble TLRs in diagnosing pneumonia necessitating hospitalization.

Biomarker	Cut-off	Index of Union	Sensitivity % (95% CI)	Specificity % (95% CI)	PPV (%)	NPV (%)	AUC % (95% CI)	*P*-value
Serum IFN-α	491.1	0.549	32.6 (20.5-47.5)	86.7 (73.8-93.7)	71.9	86.1	0.540 (0.416-0.664)	0.51
Serum IL-6	34.01	0.477	44.2 (30.4-58.9)	82.2 (68.7-90.7)	70.4	82.9	0.561 (0.437-0.686)	0.32
Serum IL-1β	910.7	0.544	41.9 (28.4-56.7)	77.8 (63.7-87.5)	64.3	78.6	0.521 (0.396-0.647)	0.73
Serum IL-4	213.9	0.521	44.2 (30.4-58.9)	77.8 (63.7-87.5)	65.5	78.6	0.552 (0.429-0.675)	0.40
Serum IL-10	155.6	0.518	51.2 (36.8-65.4)	71.1 (56.6-82.3)	62.9	72.0	0.522 (0.394-0.650)	0.73
Serum TNF-α	122.0	0.543	44.2 (30.4-58.9)	75.6 (61.3-85.8)	63.3	76.4	0.544 (0.418-0.670)	0.47
Serum TLR-2	6.995	0.476	46.5 (32.5-61.1)	80 (66.2-89.1)	69.0	80.7	0.536 (0.411-0.617)	0.56
Serum TLR-4	3.245	0.610	44.2 (30.4-58.9)	68.9 (54.3-80.5)	57.6	69.9	0.502 (0.379-0.624)	0.98
Saliva IFN-α	1.655	0.285	79.0 (56.7-91.5)	66.67 (48.8-80.8)	60.0	76.0	0.719 (0.567-0.871)	0.010
Saliva IL-6	2.305	0.290	68.4 (46.0-84.6)	76.7 (59.1-88.2)	65.0	83.8	0.742 (0.597-0.888)	0.005
Saliva IL-1β	0.8900	0.567	47.4 (27.3-68.3)	70.0 (52.1-83.3)	50.0	78.7	0.565 (0.400-0.730)	0.45
Saliva IL-10	2.665	0.218	79.0 (56.7-91.5)	73.3 (55.6-85.8)	65.2	81.3	0.772 (0.634-0.910)	0.002
Saliva TNF-α	3.665	0.165	84.2 (62.4-94.5)	73.3 (55.6-85.8)	66.7	81.3	0.833 (0.721-0.946)	<0.0001
Saliva TLR-2	1.970	0.232	84.2 (62.4-94.5)	66.7 (48.8-80.8)	61.5	76.0	0.757 (0.615-0.899)	0.003
Saliva TLR-4	2.350	0.251	79.0 (56.7-91.5)	70 (52.1-83.3)	62.5	78.7	0.768 (0.632-0.903)	0.002

PPV, positive predictive value; NPV, negative predictive value; AUC, area under curve; CI, confidence interval.

### 3.6 Correlation of clinical symptoms, laboratory parameters, and cytokines

There was a strong positive correlation between several serum cytokines, indicating coordinated inflammatory responses. Among saliva markers in inpatients, IL-1β and TNF-α showed no significant correlation with other biomarkers. However, in outpatients, TNF-α had a significant positive correlation with IL-10 in this group. Moreover, serum markers in the outpatient group did not show any significant correlations with other biomarkers. However, there was a significant positive correlation between saliva soluble TLR-2/TLR-4 levels and clinical symptoms, including feeding difficulties, loss of appetite, and restlessness. In contrast, soluble TLR-4 in inpatients exhibited a more moderate negative correlation with several serum markers. Furthermore, CD3 and CD8 consistently demonstrated moderate negative correlations with serum markers in inpatients. However, this pattern was not observed in the outpatient group (**Supplementary Figure 4**).

## 4 Discussion

TLR-2 and TLR-4 levels were significantly increased in children with lower respiratory tract infections compared to healthy controls, and these levels were even higher in patients with severe pneumonia compared to those with mild pneumonia according to data of the current study. Early recognition of pneumonia, which is still one of the most common causes of childhood deaths in the world, is very important.

One of the novel aspects of our study is the age-stratified analysis of serum cytokines, which revealed significantly elevated levels of IL-6 and TNF-α in infants aged 0 to 1 year compared to older children, suggesting that younger children may have a more robust or dysregulated innate immune response during pneumonia, potentially contributing to increased disease severity and hospitalization rates. Moreover, these results further underscore the value of non-invasive sampling methods, including saliva collection in pediatric groups, where blood sampling remain challenging. To the best of our knowledge, this is the first study to concurrently analyze soluble TLR-2 and TLR-4 in both saliva and serum in pediatric pneumonia, highlighting the diagnostic utility of saliva as a non-invasive alternative.

Although no study examining CAP and salivary TLR expression has been found in the literature, the potential role of TLRs in lung injury and pneumonia has been tried to be clarified by many researchers (15, 31–33). TLR evaluation was performed in serum and BAL samples and the interaction of these receptors with several bacteria and viruses was also investigated (34–36). There are many studies in the literature examining the relationship between TLR-2 and *S. pneumoniae.* In a mouse model, the surface lipoprotein deficient Δlgt pneumococcal mutant strain was used and TLR-2 signaling is dependent on pneumococcal lipoproteins, and macrophage NF-κB activation and TNF-α release are reduced in response to the Δlgt chain (37). Genetic deficiency of TLR signaling pathway proteins increases the risk of invasive *S. pneumoniae* infection (38). In our study, there was no remarkable change in TLR-2 response between virus-induced and other pneumonia. However, a significant increase of salivary TLR-2 levels was observed in pediatric patients with viral pneumonia compared to healthy controls.

Severe acute respiratory syndrome coronavirus 2 may also damage by activating TLRs, especially TLR-2, and may cause the secretion of pro-inflammatory cytokines independent of viral entry (39). TLR-1/2 heterodimers bound by the SARS-CoV-2 Spike (S)-protein may contribute to the hyperinflammatory state and lung injury seen in COVID-19. TLR-2 forms heterodimers with TLR-1 and TLR-6, ​​respectively, to form functional cell surface receptors. Several *in vitro* as well as and *in vivo* studies demonstrated that TLR-2 recognizes SARS-CoV-2 envelope protein and causes MyD88-induced inflammation (20). Whether the binding of these heterodimers to the S-protein affects the immune response in COVID-19 is still unclear (21). While these proteins typically cause the initiation of the signaling pathway through the MyD88 pathway, in some immune cells, TLR-2 is taken into the cell and endosomes, and consequently causes the induction of type I/III IFN responses (22). TLR-1 only acts as a heterodimer with TLR-2 and especially TLR1-7202G in sepsis contributes to the predisposition to acute respiratory distress syndrome (26) in relation to NF-κB activity and cytokine production (40). In addition, it also has a role in the regulation of pro-inflammatory pathways through T regulatory cells (41). The S-protein of SARS-CoV-2 is predicted to bind TLR-1/2; TLR-1/2 agonism may contribute to the pathophysiology of cytokine storm, and secondary lung injury seen in severe COVID-19 patients (21). In the current study, no significant difference was observed in serum TLR-2 levels between pediatric patients with COVID-19 pneumonia and those in the control group. In the lung, TLR-4 expression has been demonstrated in alveolar and bronchial epithelial and vascular endothelial cells (3, 16, 32). In the study by Siebert et al. (42), it was thought that TLR-2 upregulation was particularly present in children with lower respiratory tract infections, which may reflect recurrent episodes of pneumococcal infection over the years. It has been observed that LPS and TLR-4-dependent IL-6 levels are higher in children with recurrent pneumococcal pneumonia than in healthy children (42). Similarly, in our results, both TLR-4 and IL-6 upregulation were revealed in saliva samples obtained from children with lower respiratory tract infections. However, high pneumococcal colonization was not demonstrated. Furthermore, Shinya et al. (35) demonstrated the association between TLR-4 and viral diseases, showing that pre-stimulation of the TLR-4 pathway with LPS protected mice from lethal infection with the H5N1 influenza virus (35). Using the pathway of TLR-4 knockout mice, TLR-4/TRIF pathway is required for this protective effect and TLR-4/TRIF axis, having an important role in stimulating the protective innate immunity against H5N1 influenza A virus infection. In a further study, the stimulation of TLR-4 signaling by multiple viral and bacterial pathogens was important for acute lung injury (31). While influenza virus is recognized by multiple pattern recognition receptors, including TLR-3, TLR-7, TLR-8, and TLR-10, it is thought that a multiple inflammation pathway mainly based on TLR-4 in this damage (31). Unlike these studies, influenza virus was not detected as a causative pathogen at the time of the current study period (pandemic period), whereas human rhinovirus, RSV-A and RSV-B are the main causative viruses during the study period.

Although the prototypical TLR-4 ligand is LPS, other ligands have also been identified, including glycolipids, viral structural proteins, and damage-associated molecular patterns (43, 44). It has been suggested that viral structural proteins and glycolipids in COVID-19 are pathogen-associated molecular patterns (PAMPs) that initiate an innate immune response and bind TLR-4. According to the data by Zhao et al., purified SARS-CoV-2 S protein binds TLR-4 with strong affinity *in vitro* and induces TLR-4-dependent IL-1β response, similar to the response observed with LPS (43). Activation of TLR-4 by S protein is thought to increase the expression of angiotensin converting enzyme 2 and further increase viral infectivity by leading to an autocrine cycle (45). In addition, when SARS-CoV-2 binds to TLR-4, it activates transcription factors such as activator protein-1 (AP-1), NF-kB and IRF (46). TLR-4 has also been found to regulate IL-6 through NF-kB (47). Several other research studies have revealed involvement of TLR-4 in SARS-CoV-2 through molecular insertion study revealing the interaction between S protein and cell surface TLRs (48, 49). The interaction of the S protein of SARS-CoV-2 with TLR-4 was found to be higher than that of other TLRs. However, the reason behind such strong recognition of TLR-4 by SARS-COV-2 is not fully understood (46). According to the results of a study conducted on 48 Korean patients, TLR-4-mediated activation of the NF-κB signaling pathway was shown to play a role in the hyperinflammatory responses in COVID-19 patients, suggesting that TLR-4 signaling has a significant role in the pathogenesis of the disease. The increase in IL-1β and all downstream signaling molecules also resulted in uncontrolled pathological inflammation (50). In our study, no significant difference was found in TLR-4 expression in children with COVID-19 pneumonia compared to healthy controls. Interestingly, there was a different interaction of TLRs with viruses; for instance, serum TLR-2 and TLR-4 levels were significantly different in cases infected with human rhinovirus and SARS-CoV-2 in addition to studied cytokines. These results made us hypothesize that many viral agents use similar pathogenetic mechanisms, but the main factor contributing to pathological processes in COVID-19 patients may be affected by other biomarkers such as chemokines, as we have shown in our previous studies (51).

Tumor necrosis factor -α is secreted by activated monocytes/macrophages in response to a variety of stimuli, including LPS, viruses, or gram-positive or gram-negative bacteria. It can also be secreted by activated B or T cells, and natural killer (NK) cells. Patients receiving anti-TNF-α therapy are at risk for lower respiratory tract infections (52). It is known that TNF-α has an important role in pulmonary homeostasis. In our study, TNF-α was found to be higher in saliva samples obtained from hospitalized patients with severe pneumonia compared to the control group. Moreover, in our study, a significant increase was found in IL-6 and IL-10 levels in severe pneumonia patients as compared to the controls, but the similar increase was not observed in IL-1β levels. Haugen et al. stated that IL-1β, IL-6 and TNF-α levels were found to be high in pediatric patients diagnosed with CAP, but no significant difference was found in IL-10 levels (53). In previous reports, especially IL-6 and IL-10 were significantly associated with different severity criteria such as mental confusion, hypotension, pleural effusion and bacteremia in patients with a diagnosis of CAP (54, 55). In adults with COVID-19, mRNA expression of pro-inflammatory cytokines (IL-1β, IL-6, and TNF-α) were elevated in both saliva and plasma in mild and severe disease, and their levels correlated with disease severity (56). Menèndez et al. (57) reported in an adult study that while IL-10 level is higher, TNF-α level is lower in pneumonia caused by influenza virus compared to bacterial causes. In our study, it was shown that IL-10 and TNF-α cytokine levels were higher in both serum and saliva samples of pediatric patients with viral pneumonia due to viruses, particularly human rhinovirus as compared to SARS-CoV-2.

C-reactive protein and PCT continue to maintain their importance as old and classical biomarkers according to the findings of the present study. However, these markers do not provide information about the etiological cause. Many studies in pediatric CAP have shown that high CRP levels are associated with adverse outcomes such as lobar consolidation, fever, prolonged hospital stay, pleural effusion, and even death (58). Our data similarly showed that children with low CRP levels had a lower risk of serious pneumonia, raising the possibility of using this marker to predict severity at the time of emergency department admission. In addition, PCT was found to be superior to CRP in severity assessment. In a publication, an increase in both PCT and CRP values ​​in lobar CAP compared to bronchopneumonia was reported, but PCT was found to have better sensitivity and specificity in detecting CAP in correlation with radiological findings (59). However, there are also publications where the opposite is demonstrated and the superiority of PCT over CRP is not evident (60). According to our results, similar to many publications, classical markers such as CRP and PCT still guide us in determining the severity of pneumonia.

While the findings of this study are valuable, several limitations may affect the generalizability of the results and should be acknowledged. The first of these is the low case numbers of upper and lower respiratory tract infections in children due to the less transmission of causative agents due to the prevention measures during COVID-19 pandemic. In addition, the study was performed at a single center, which may limit the applicability of the findings to broader populations (e.g., through Europe or world). Moreover, admissions to the hospitals have been affected by the fear of the SARS-CoV-2 virus. Secondly, with the significant increase of the MIS-C cases, the presence of patients developing multiorgan failure during the disease course of severe COVID-19 has caused difficulties in distinguishing patients diagnosed with COVID-19 pneumonia and MIS-C. Therefore, such kind of cases in addition to MIS-C cases have also been excluded from the study to understand the exact TLR profile in CAP. Third, saliva samples could not be collected from SARS-CoV-2-positive patients due to strict infection control protocols prohibiting aerosol-generating procedures. This restriction prevented virus-specific comparison of local airway immune responses in SARS-CoV-2 pneumonia. However, this did not compromise our primary severity-based analyses, which included bacterial and non-SARS-CoV-2 viral pneumonias, nor our serum-based virus-specific comparisons.

It is necessary to identify the most accurate and fastest biomarkers in the diagnosis of CAP in pediatric patients. Considering the difficulty of obtaining serum samples from pediatric patients with this study, the importance of using a material such as saliva that can be obtained easily and painlessly, especially in the diagnosis of CAP, has been demonstrated in the current study. In addition, our study sheds light on future studies which have the potential to evaluate the effects of new agonist and antagonist drugs work on TLR-pathway in pediatric patients with CAP, which were previously limited to adult studies and animal experiments.

## Data Availability

The raw data supporting the conclusions of this article will be made available on reasonable request.

## Supplementary material

Supplementary Figure 1 Participant enrollment and biospecimen collection. Flowchart showing the distribution of serum and saliva samples collected from hospitalized patients (*n* = 43), outpatients (*n* = 24), and healthy controls (*n* = 22). Sample availability varied as not all participants provided both specimen types.

Supplementary Figure 2 Age-stratified serum cytokine and TLR profiles within clinical cohorts. Each panel depicts one cytokine or TLR; y-axes are scaled independently. Boxplots display the median (center line), interquartile range (box), and whiskers extending to 1.5×IQR; points are individual participants. Colors denote age categories: 0 to 1 yr (green), 1 to 5 yr (blue) (32), and 6 to 18 yr (32). For each cytokine, a two-way ANOVA (factors: age group and cohort—Control, Inpatient, Outpatient) was followed by Tukey’s HSD for pairwise contrasts. Significant age-group differences within the same cohort are marked above the corresponding box with asterisks (**p* < 0.05, ***p* < 0.01, ****p* < 0.001; Tukey-adjusted). Absence of asterisks indicates non-significance.

Supplementary Figure 3 Age-stratified saliva cytokine and TLR profiles within clinical cohorts. Each panel depicts one cytokine or TLR; y-axes are scaled independently. Boxplots display the median (center line), interquartile range (box), and whiskers extending to 1.5×IQR; points are individual participants. Colors denote age categories: 0 to 1 yr (green), 1 to 5 yr (blue) (32), and 6 to 18 yr (32). Because several *age group × cohort* cells contained fewer than five observations, formal hypothesis testing was not undertaken; the figure was provided for qualitative comparison only.

Supplementary Figure 4 The correlation matrix of patients with pneumonia provides a comprehensive view of the relationships between clinical symptoms, lab values, lymphocyte subsets, serum and saliva cytokines, and soluble TLRs in inpatients and outpatients separately. The numbers within the boxes represent significant Pearson correlation coefficients (r values), indicating the strength and direction of the relationships between various biomarkers. Empty boxes indicate no significant correlation between the markers.
