# Supplementary figures and images for: The role of soluble toll-like receptor-2 and 4 in children with pneumonia: a combined analysis of saliva and serum samples
Source: Front Immunol. 2026 Feb 13;17:1657027. doi: 10.3389/fimmu.2026.1657027 (PMC12945803; doi:10.3389/fimmu.2026.1657027)

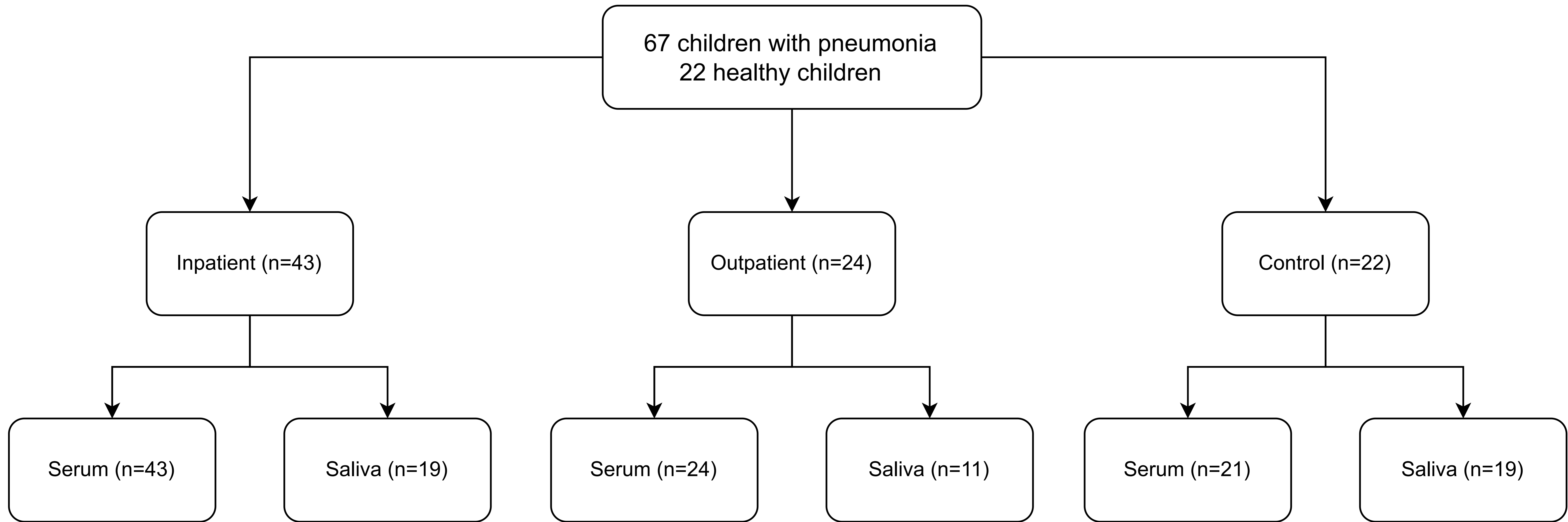

Supplement: Supplementary Figure 1 — Participant enrollment and biospecimen collection. Flowchart showing the distribution of serum and saliva samples collected from hospitalized patients (n = 43), outpatients (n = 24), and healthy controls (n = 22). Sample availability varied as not all participants provided both specimen types. [file DataSheet1.pdf]

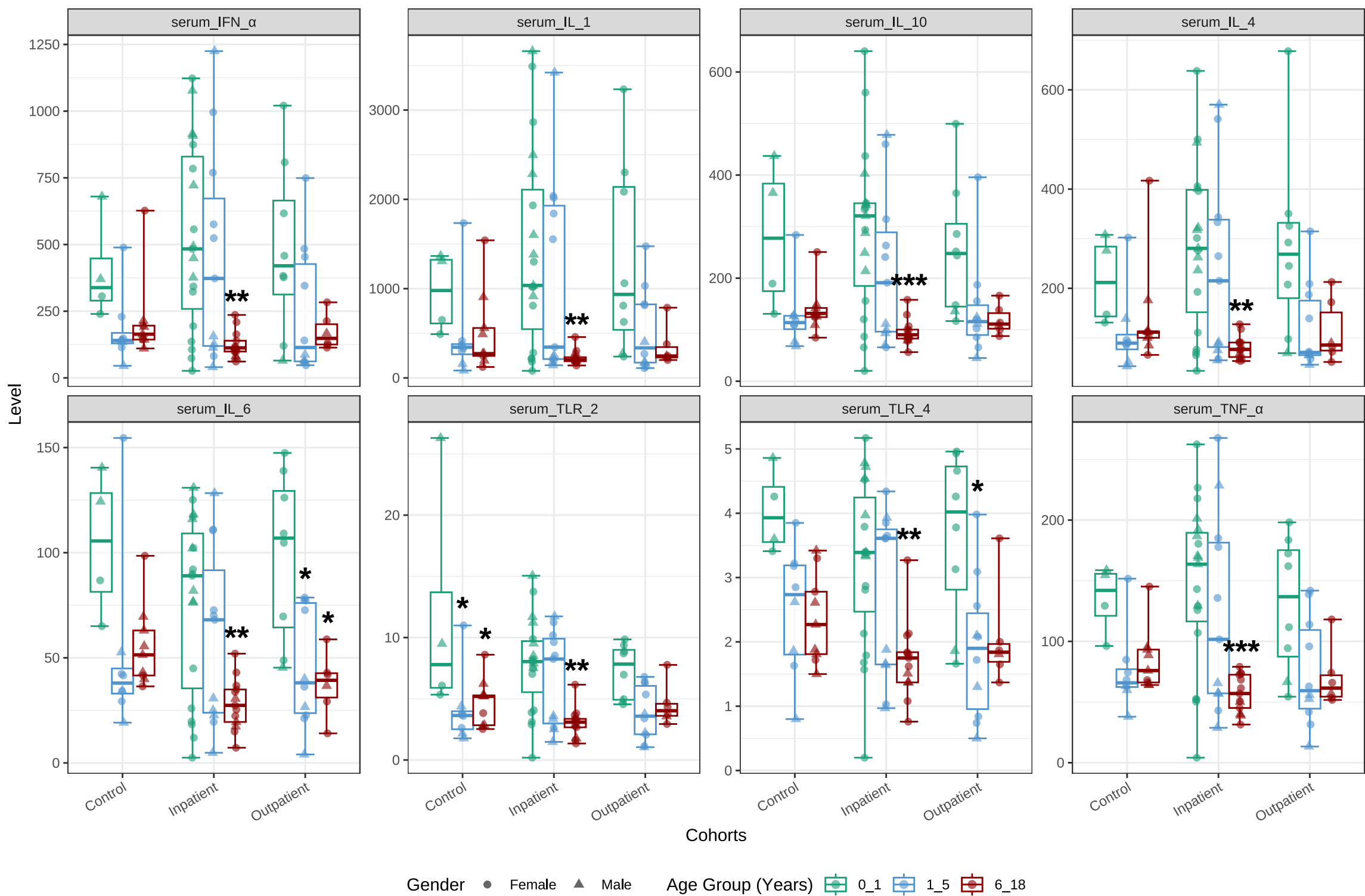

Supplement: Supplementary Figure 2 — Age-stratified serum cytokine and TLR profiles within clinical cohorts. Each panel depicts one cytokine or TLR; y-axes are scaled independently. Boxplots display the median (center line), interquartile range (box), and whiskers extending to 1.5×IQR; points are individual participants. Colors denote age categories: 0 to 1 yr (green), 1 to 5 yr (blue) (32), and 6 to 18 yr (32). For each cytokine, a two-way ANOVA (factors: age group and cohort—Control, Inpatient, Outpatient) was followed by Tukey’s HSD for pairwise contrasts. Significant age-group differences within the same cohort are marked above the corresponding box with asterisks (*p < 0.05, **p < 0.01, ***p < 0.001; Tukey-adjusted). Absence of asterisks indicates non-significance. [file DataSheet2.pdf]

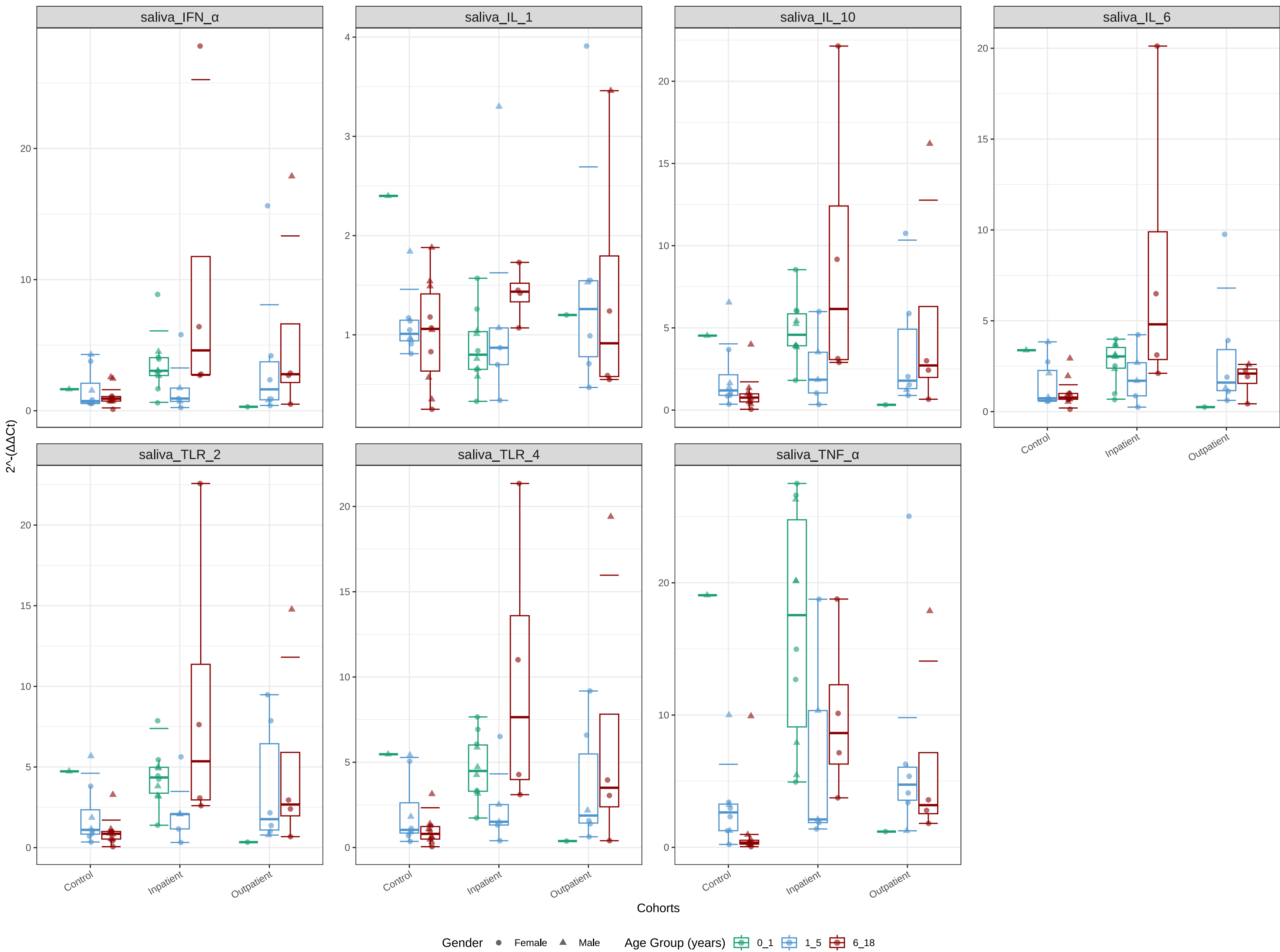

Supplement: Supplementary Figure 3 — Age-stratified saliva cytokine and TLR profiles within clinical cohorts. Each panel depicts one cytokine or TLR; y-axes are scaled independently. Boxplots display the median (center line), interquartile range (box), and whiskers extending to 1.5×IQR; points are individual participants. Colors denote age categories: 0 to 1 yr (green), 1 to 5 yr (blue) (32), and 6 to 18 yr (32). Because several age group × cohort cells contained fewer than five observations, formal hypothesis testing was not undertaken; the figure was provided for qualitative comparison only. [file DataSheet3.pdf]
