# Supplementary material for: The role of soluble toll-like receptor-2 and 4 in children with pneumonia: a combined analysis of saliva and serum samples
Source: Front Immunol. 2026 Feb 13;17:1657027. doi: 10.3389/fimmu.2026.1657027 (PMC12945803; doi:10.3389/fimmu.2026.1657027)
Supplement: Supplementary file 5 [file Table1.docx]

**Supplementary Table 1A.** The protocol of the experiment for saliva

| **Step** | **Action** | **Reagent / Volume** | **Conditions** | **Notes*** |
| --- | --- | --- | --- | --- |
| 1 | Aliquot saliva to tube | Saliva, 150 µl → 2 ml tube | — | — |
| 2 | Adjust volume, mix | PBS (pH 7.4), 100 µl (final 250 µl) | Gentle mix | — |
| 3 | Lyse | Buffer RL, 250 µl | Mix a few seconds | — |
| 4 | Add alcohol | 96–100% ethanol, 250 µl | Gentle mix | — |
| 5 | Bind RNA (load) | Transfer 600 µl lysate to spin column | 3,500 × g, 1 min | If not all passes → Step 6 |
| 6 | Complete binding (if needed) | — | 14,000 × g, 1 min | Optional extra spin |
| 7 | Discard flow-through | — | — | Keep column in collection tube |
| 8 | Wash #1 | Wash Solution A, 400 µl | 14,000 × g, 1 min | If liquid remains, extend spin |
| 9 | Wash #2 | Wash Solution A, 400 µl | 14,000 × g, 1 min | Repeat as above |
| 10 | Move column | Place column in clean 1.5 ml tube | — | — |
| 11 | Elute RNA | Elution Solution A, 50 µl | 200 × g, 2 min | Pre-wet step per protocol |
| 12 | Elute RNA (collect) | — | 14,000 × g, 1 min | Collect eluate (RNA) |

***One hundred fifty µl of saliva sample was taken into a 2 ml microcentrifuge tube. The final volume was adjusted to 250 µl by adding 100 µl of pH 7.4 phosphate buffered saline solution. Then, 250 µl of Buffer RL was added and mixed thoroughly for a few seconds and 250 µl of 96-100% ethanol was added and gently mixed thoroughly. 600 µl of the prepared lysate was transferred to the spin column and centrifuged at 3500 x g for 1 min.. If all the lysate did not pass through the column, it was centrifuged for 1 more min. at 14,000 x g. The supernatant that passed through the column was discarded and 400 µl wash solution A was added to the column and centrifuged at 14.000 x g for 1 min.. This process was repeated twice. If all the liquid did not pass through the column, the centrifugation time was extended by a few min.. The waste tube was discarded and a sterile 1.5 ml microcentrifuge tube from which RNA was to be obtained was attached to the column. 50 µl of elution solution A was added to the column and centrifuged at 200 x g for 2 min.. Then RNA was obtained by centrifugation at 14,000 x g for 1 min.. A brief mix and centrifugation were performed for Sensifat (Bioline) cDNA Kit Reverse Transcriptase process. Then, ABI Veriti 96 device was used for reverse transcription. The device operates according to a protocol that includes the steps of reverse transcription at 25°C for 10 min. of primer binding, 15 min. of reverse transcription at 42°C, and 5 min. of enzyme inactivation at 85°C. At the end of the reaction, the enzyme was inactivated at 85°C for 5 min., and the cDNA products were stored at -20°C for a long time. The preparation was according to the manufacturers’ instructions.

**Supplementary Table 1B.** Primer sequences which used in RT-PCR

| **Oligonucleotide name** | **Oligonucleotide sequence (5’🡪3’)** | **Optimum Temperature** |
| --- | --- | --- |
| TLR2-hsa-EJ-F | TGGTAGTTGTGGGTTGAA | 61℃ |
| TLR2-hsa-EJ-R | CTTGGAGAGGCTGATGAT |  |
| IL1B-hsa-EJ-F | GGCTTATTACAGTGGCAATG | 61℃ |
| IL1B-hsa-EJ-R | TAGTGGTGGTCGGAGATT |  |
| IL4-hsa-EJ-F | CCTCTGTTCTTCCTGCTA | 59℃ |
| IL4-hsa-EJ-R | TCTCAGTTGTGTTCTTGGA |  |
| IL10-hsa-EJ-F | TCAGCAGAGTGAAGACTT | 63℃ |
| IL10-hsa-EJ-R | ACCCAGGTAACCCTTAAAG |  |
| TLR4-hsa-EJ-F | TTATCACGGAGGTGGTTC | 59℃ |
| TLR4-hsa-EJ-R | TGGATTTCACACCTGGAT |  |
| TNF-hsa-EJ-F | CTTGTTCCTCAGCCTCTT | 61.5℃ |
| TNF-hsa-EJ-R | TCGAGAAGATGATCTGACTG |  |
| IL6-hsa-Algn-F | AGCAAAGAGGCACTGGCAGAA | 56℃ |
| IL6-hsa-Algn-R | CACCAGGCAAGTCTCCTCATTGAA |  |
| IFNA1-hsa-Algn-F | AAGGAGGAAGGAATAACA | 53℃ |
| IFNA1-hsa-Algn-R | TATCAGCATGGTCATAGT |  |
| ACTB-hsa-F | TGAAGATCAAGATCATTG | 59℃ |
| ACTB-hsa-R | TAACGCAACTAAGTCATA |  |
