# Supplementary material for: The role of soluble toll-like receptor-2 and 4 in children with pneumonia: a combined analysis of saliva and serum samples
Source: Front Immunol. 2026 Feb 13;17:1657027. doi: 10.3389/fimmu.2026.1657027 (PMC12945803; doi:10.3389/fimmu.2026.1657027)
Supplement: Supplementary file 6 [file Table2.docx]

**Supplementary Table 2.** Laboratory investigation of patients with pneumonia

| **Laboratory values** | **Inpatient**  *n*=43*^1^* | **Outpatient**  *n*=24*^1^* | ***p*-value***^2^* |
| --- | --- | --- | --- |
| Hemoglobin (g/dL) | 12.30 (11.15 - 13.70) | 13.25 (12.50 - 13.93) | 0.064 |
| White blood cell (4,000-13,800 /μl) | 11,000 (6,950 - 14,750) | 10,800 (8,550 - 13,600) | 0.5 |
| Absolute Lymphocyte Count (1,300-5,800 /μl ) | 2,680 (1,575 - 3,820) | 2,855 (2,400 - 4,128) | 0.3 |
| Absolute Neutrophil Count (1,600-8,300 /μl) | 6,290 (4,035 - 9,045) | 5,750 (4,443 - 9,473) | 0.7 |
| Neutrophil- Lymphocyte Ratio | 2.31 (1.59 - 4.42) | 2.45 (1.40 - 3.85) | 0.7 |
| Platelet count (189-394 x 10^9^/L) | 316 (214 - 381) | 313 (237 - 368) | >0.9 |
| ESR (mm/h) | 13 (5 - 27) | 5 (2 - 17) | 0.053 |
| C-reactive protein (<0.5 mg/dL) | 2.7 (0.7 - 7.3) | 0.7 (0.6 - 1.3) | 0.003 |
| Procalcitonin (<0.1 ng/mL) | 0.2 (0.1 - 0.8) | 0.1 (0.0 - 0.1) | 0.007 |
| IL-6 (<6.4 pg/mL) | 48 (29 - 72) | NA (NA - NA) |  |
| **Lymphocyte Subsets** |  |  |  |
| CD3^+^ cells/μl, % | 60 (50 - 71) | 65 (60 - 71) | 0.2 |
| CD4^+^ cells/μl, % | 34 (24 - 44) | 39 (32 - 42) | 0.2 |
| CD8^+^ cells/μl, % | 20 (15 - 29) | 23 (18 - 26) | 0.5 |
| CD16^+^ CD56^+^ cells/μl, % | 9.0 (7.0 - 12.5) | 10.0 (8.0 - 13.0) | 0.6 |
| CD19^+^ cells/μl, % | 26 (17 - 39) | 21 (16 - 25) | 0.094 |
| *^1^*Median (IQR)  *^2^*Wilcoxon rank sum test | | | |
