# Supplementary material for: The role of soluble toll-like receptor-2 and 4 in children with pneumonia: a combined analysis of saliva and serum samples
Source: Front Immunol. 2026 Feb 13;17:1657027. doi: 10.3389/fimmu.2026.1657027 (PMC12945803; doi:10.3389/fimmu.2026.1657027)
Supplement: Supplementary file 7 [file Table3.docx]

**Supplementary Table 3.** Laboratory investigation of inpatients who had recurrent LTRI and non-recurrent LTRI.

| **Laboratory values** | **Non-Recurrent LTRI**  *n*=25*^1^* | **Recurrent LTRI**  *n*=18*^1^* | ***p*-value***^2^* |
| --- | --- | --- | --- |
| **Age** (year) | 2.0 (0.0 - 9.0) | 2.0 (1.0 - 3.0) | 0.8 |
| **Age groups** |  |  | 0.3 |
| *Preschool (0-5 years)* | 15 (60.00%) | 15 (83.33%) |  |
| *School (6-12 years)* | 6 (24.00%) | 2 (11.11%) |  |
| *Adolescent (13-18 years)* | 4 (16.00%) | 1 (5.56%) |  |
| **Gender (Male)** | 8 (32.00%) | 7 (38.89%) | 0.9 |
| Hemoglobin (g/dL) | 12.40 (11.00 - 14.20) | 12.15 (11.30 - 13.70) | 0.9 |
| White blood cell (4,000-13,800 /μl ) | 9,100 (6,800 - 13,700) | 11,300 (8,000 - 15,600) | 0.3 |
| Absolute Lymphocyte Count (1,300-5,800 /μl) | 2,480 (1,390 - 3,460) | 2,850 (1,790 - 4,180) | 0.4 |
| Absolute Neutrophil Count (1,600-8,300 /μl) | 4,400 (3,680 - 8,770) | 6,550 (5,350 - 10,820) | 0.12 |
| Platelet count (189-394 x 10^9^/L ) | 325 (242 - 381) | 280 (207 - 352) | 0.4 |
| Neutrophil- Lymphocyte Ratio | 2.31 (1.57 - 4.32) | 2.25 (1.75 - 5.40) | 0.8 |
| ESR (mm/h) | 13 (5 - 24) | 13 (6 - 33) | 0.6 |
| C-reactive protein (<0.5 mg/dL) | 3 (1 - 8) | 2 (1 - 6) | 0.6 |
| Procalcitonin (<0.1 ng/mL) | 0 (0 - 1) | 0 (0 - 0) | >0.9 |
| IL-6 (<6.4 pg/mL) | 30 (28 - 81) | 55 (48 - 63) | 0.9 |
| **Lymphocyte Subsets** |  |  |  |
| CD3^+^ cells/μl, % | 65 (50 - 72) | 58 (50 - 70) | >0.9 |
| CD4^+^ cells/μl, % | 33 (23 - 42) | 35 (26 - 45) | 0.6 |
| CD8^+^ cells/μl, % | 24 (16 - 29) | 18 (14 - 25) | 0.14 |
| CD16^+^ CD56^+^ cells/μl, % | 10.0 (7.0 - 13.0) | 9.0 (6.0 - 11.0) | 0.2 |
| CD19^+^ cells/μl, % | 26 (15 - 40) | 30 (17 - 37) | 0.7 |
| *^1^n* (%); Median (Q1 - Q3)  *^2^*Wilcoxon rank sum test; Wilcoxon rank sum exact test | | | |
