# Supplementary material for: The role of soluble toll-like receptor-2 and 4 in children with pneumonia: a combined analysis of saliva and serum samples
Source: Front Immunol. 2026 Feb 13;17:1657027. doi: 10.3389/fimmu.2026.1657027 (PMC12945803; doi:10.3389/fimmu.2026.1657027)
Supplement: Supplementary Figure 4 — The correlation matrix of patients with pneumonia provides a comprehensive view of the relationships between clinical symptoms, lab values, lymphocyte subsets, serum and saliva cytokines, and soluble TLRs in inpatients and outpatients separately. The numbers within the boxes represent significant Pearson correlation coefficients (r values), indicating the strength and direction of the relationships between various biomarkers. Empty boxes indicate no significant correlation between the markers. [file DataSheet4.pdf]

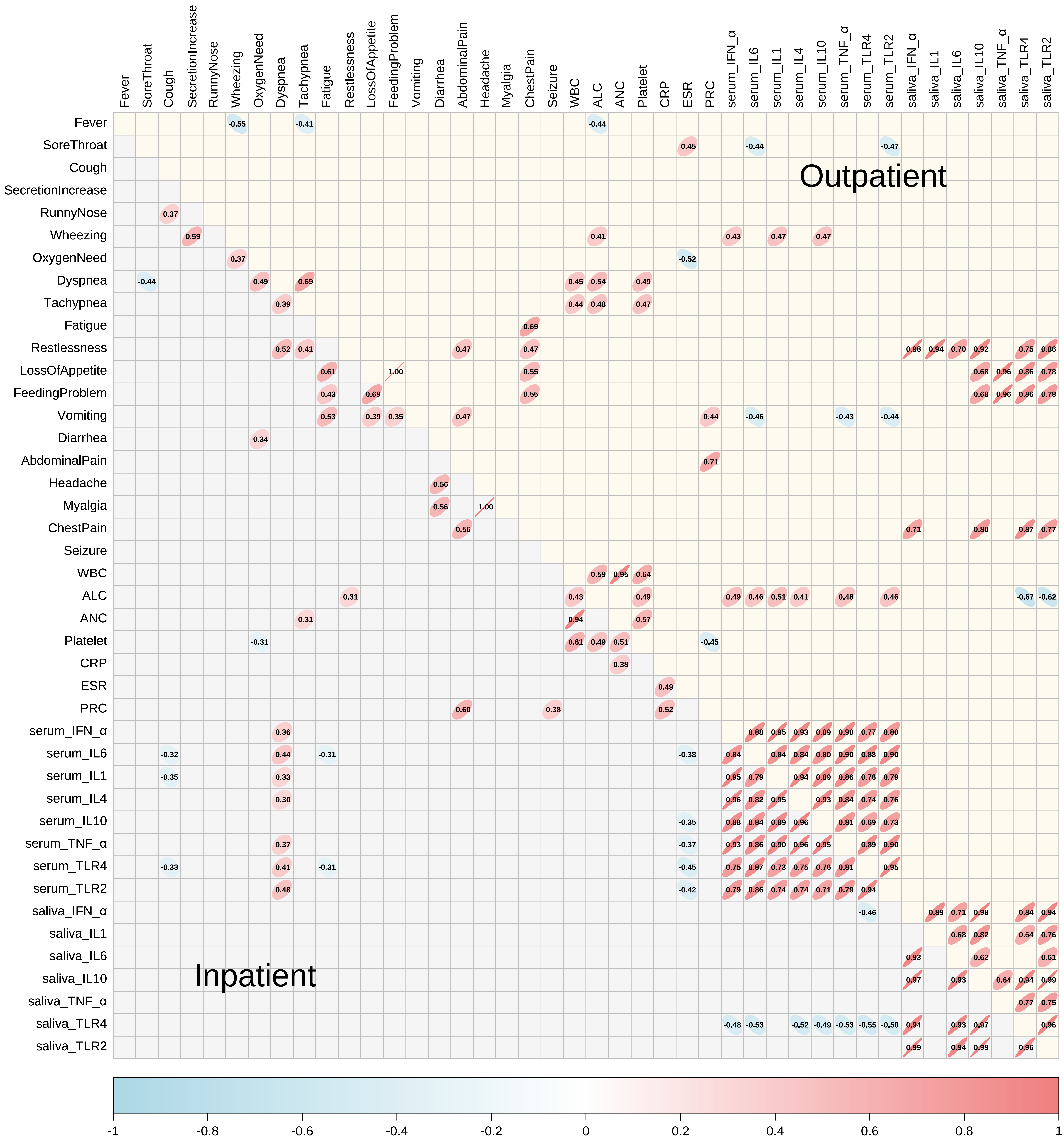
Fever
SoreThroat
Cough
SecretionIncrease
RunnyNose
Wheezing
OxygenNeed
Dyspnea
Tachypnea
Fatigue
Restlessness
LossOfAppetite
FeedingProblem
Vomiting
Diarrhea
AbdominalPain
Headache
Myalgia
ChestPain
Seizure
WBC
ALC
ANC
Platelet
CRP
ESR
PRC
serum_IFN_α
serum_IL6
serum_IL1
serum_IL4
serum_IL10
serum_TNF_α
serum_TLR4
serum_TLR2
saliva_IFN_α
saliva_IL1
saliva_IL6
saliva_IL10
saliva_TNF_α
saliva_TLR4
saliva_TLR2
Outpatient
Inpatient
-1
-0.8
-0.6
-0.4
-0.2
0
0.2
0.4
0.6
0.8
1
